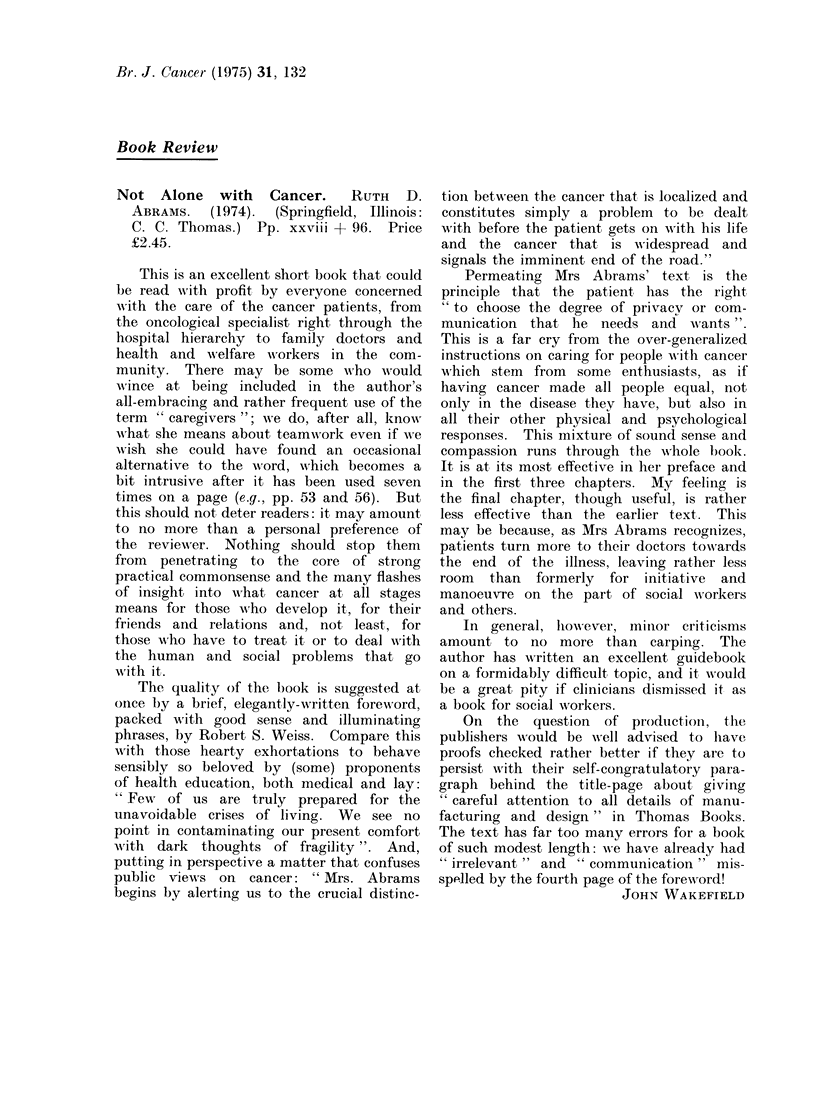# Not Alone with Cancer

**Published:** 1975-01

**Authors:** John Wakefield


*Br. J. Cancer* (1975) **31**, 132

### *Book Review*

**Not Alone with Cancer.** RUTH D. ABRAMS. (1974). (Springfield, Illinois: C. C. Thomas.) Pp. xxviii + 96. Price £2.45.

This is an excellent short book that could be read with profit by everyone concerned with the care of the cancer patients, from the oncological specialist right through the hospital hierarchy to family doctors and health and welfare workers in the community. There may be some who would wince at being included in the author's all-embracing and rather frequent use of the term " caregivers "; we do, after all, know what she means about teamwork even if we wish she could have found an occasional alternative to the word, which becomes a bit intrusive after it has been used seven times on a page (*e.g.*, pp. 53 and 56). But this should not deter readers: it may amount to no more than a personal preference of the reviewer. Nothing should stop them from penetrating to the core of strong practical commonsense and the many flashes of insight into what cancer at all stages means for those who develop it, for their friends and relations and, not least, for those who have to treat it or to deal with the human and social problems that go with it.

The quality of the book is suggested at once by a brief, elegantly-written foreword, packed with good sense and illuminating phrases, by Robert S. Weiss. Compare this with those hearty exhortations to behave sensibly so beloved by (some) proponents of health education, both medical and lay: " Few of us are truly prepared for the unavoidable crises of living. We see no point in contaminating our present comfort with dark thoughts of fragility ". And, putting in perspective a matter that confuses public views on cancer: " Mrs. Abrams begins by alerting us to the crucial distinction between the cancer that is localized and constitutes simply a problem to be dealt with before the patient gets on with his life and the cancer that is widespread and signals the imminent end of the road."

Permeating Mrs Abrams' text is the principle that the patient has the right " to choose the degree of privacy or communication that he needs and wants ". This is a far cry from the over-generalized instructions on caring for people with cancer which stem from some enthusiasts, as if having cancer made all people equal, not only in the disease they have, but also in all their other physical and psychological responses. This mixture of sound sense and compassion runs through the whole book. It is at its most effective in her preface and in the first three chapters. My feeling is the final chapter, though useful, is rather less effective than the earlier text. This may be because, as Mrs Abrams recognizes, patients turn more to their doctors towards the end of the illness, leaving rather less room than formerly for initiative and manoeuvre on the part of social workers and others.

In general, however, minor criticisms amount to no more than carping. The author has written an excellent guidebook on a formidably difficult topic, and it would be a great pity if clinicians dismissed it as a book for social workers.

On the question of production, the publishers would be well advised to have proofs checked rather better if they are to persist with their self-congratulatory paragraph behind the title-page about giving " careful attention to all details of manufacturing and design " in Thomas Books. The text has far too many errors for a book of such modest length: we have already had " irrelevant " and " communication " misspelled by the fourth page of the foreword!

JOHN WAKEFIELD